# Bevacizumab-Controlled Delivery from Polymeric Microparticle Systems as Interesting Tools for Pathologic Angiogenesis Diseases

**DOI:** 10.3390/polym14132593

**Published:** 2022-06-26

**Authors:** Giulia De Negri Atanasio, Pier Francesco Ferrari, Roberta Campardelli, Giuseppe Firpo, Patrizia Perego, Domenico Palombo

**Affiliations:** 1 Department of Civil, Chemical and Environmental Engineering, University of Genoa, via Opera Pia, 15, 16145 Genoa, Italy; giulia.denegriatanasio@edu.unige.it (G.D.N.A.); p.perego@unige.it (P.P.); 2 Department of Physics, University of Genoa, via Dodecaneso, 33, 16146 Genoa, Italy; giuseppe.firpo@unige.it; 3 Research Center for Biologically Inspired Engineering in Vascular Medicine and Longevity, University of Genoa, via Montallegro, 1, 16145 Genoa, Italy; domenico.palombo@unige.it; 4 Department of Surgical and Integrated Diagnostic Sciences, University of Genoa, viale Benedetto XV, 6, 16132 Genoa, Italy; 5 Vascular and Endovascular Surgery Unit, IRCCS Ospedale Policlinico San Martino, largo Rosanna Benzi, 10, 16132 Genoa, Italy

**Keywords:** oncology, cardiovascular diseases, biodegradable polymers, monoclonal antibodies, drug release kinetics

## Abstract

This work is a comparative study among three different biocompatible and biodegradable polymers, poly(lactic-*co*-glycolic acid), poly(ε-caprolactone), and poly(lactic acid), used to produce microparticles for the encapsulation of bevacizumab for drug delivery purposes. All the formulations were produced using the double emulsion water-oil-water evaporation method and characterized in terms of particle mean diameter, particle size distribution, and bevacizumab entrapment efficiency. Bevacizumab cumulative release was taken into consideration to study the dissolution kinetics from the three different polymeric delivery platforms for a period of 50 days at 37 °C in phosphate buffered saline and mathematical models of the drug release kinetic were attempted in order to describe the release phenomena from the different types of the studied microparticles. Finally, cell viability on human endothelial cell line EA.hy926 was studied to define the maximum cytocompatible concentration for each microsystem, registering the mitochondrial functionality through MTS assay.

## 1. Introduction

Pathological angiogenesis is observed in several diseases, including ophthalmic disorders, cancer, and cardiovascular pathologies. Recent studies have proposed the use of bevacizumab (BEV) for a potential therapeutic effect in the treatment of atheroma progression by inhibiting neovascularization [1,2]. BEV is a recombinant humanized monoclonal antibody which acts against human vascular endothelial growth factor A (VEGF-A) in all its isoforms and in its bioactive proteolytic fragments. It is characterized by a short half-life and, for this reason, frequent injections are generally required to have a pharmacological effect. As a protein, BEV exhibits a complex 3D structure, physicochemical instability, and susceptibility to environmental factors. For all these reasons, its encapsulation can provide both the advantage of reduced administration and molecule protection from denaturing agents [3]. Moreover, its hydrophilicity, its high molecular weight, and its stability in biological fluids have limited the success of anti-VEGF therapy through BEV application. These limitations can be overcome by the use of drug delivery systems.

BEV encapsulation in micro- and nanoparticles have been attempted using biodegradable polymers, both synthetic and natural, and hydrogels.

Among biodegradable synthetic polymers, polyesters, such as poly(lactic-*co*-glycolic acid) (PLGA), poly(ε-caprolactone) (PCL), and poly(lactic acid) (PLA), have been widely explored for biomedical applications [4,5,6,7].

PLGA is a largely studied polymer in the biomedical field for its high biocompatibility, its high biodegradability, its high safety and it is approved for human parenteral use by the Food and Drug Administration (FDA) [8]. PLGA is hydrolyzed into two monomers, lactic acid and glycolic acid, which are also products of various endogenous metabolic pathways and are not associated with significant toxicity. For these reasons, it represents the most widely used biopolymer for controlled drug delivery applications [9,10].

PCL is a polymer with low biotoxicity, which means that this material has not the ability to affect the normal physiology of the system into which it is introduced, and good biocompatibility, to be intended as the ability to perform its activities without exhibiting undesirable local or systemic effects and, once in contact with cells and tissues, undesirable properties [11]. The acceptable solubility, the low melting point (59–64 °C), and the huge compatibility with different drugs have made this polymer a new tool in the drug delivery field [12].

PLA is a FDA-approved polymer for human use as implants and in formulations for sustained drug delivery [13]. It is a lipophilic and biodegradable polymer made by monomers of lactic acid, which is not related to any significant toxicity event. PLA-based particles are largely used in drug delivery and biomedical applications. An advantage in the use of this polymer is its elasticity, which provides easy control of some of its properties, such as shape and size [14].

Among natural molecules, different attempts were based on BEV encapsulation in liposomes [15]. Different pros and cons are reported in the literature, taking into account the polymer-based and the lipid-based systems. In detail, the former system presents higher encapsulation efficiency and a more sustained release over time in comparison to the latter ones [16].

The multiple emulsion solvent evaporation technique and Bangham method are often used for the preparation of micro- and nanoparticles based on synthetic polymers and lipids, respectively. These methods have several advantages, such as an appropriate control of the generated droplets and particle size distribution, an easy definition of operative parameters, and an intrinsic potentiality regarding the scale-up of the process [17,18]. For hydrogels, in addition, a cross-linking process is adopted to produce the gelation of the microstructure. This process presents the pros to be a very easy procedure performed by using green solvents, showing as cons difficulties in controlling particle dimension and drug entrapment efficiency [19].

Polyester-based nanoparticles for the encapsulation of monoclonal antibodies have also been studied in the literature [20]. It is reported that polyester-based nanoparticles for monoclonal antibody delivery provide decreased antibody dosage, increased antibody stability and protection, and present a longer therapeutic action, ultimately translating to an increased therapeutic index. PLGA microparticles (MPs) loaded with BEV have already been produced for ocular applications by Ye et al. [21]. In this study, loaded microspheres were produced by a modified double emulsion technique, named solid-in-oil-in-hydrophilic oil method. Once produced, the particles were characterized by a mean diameter of about 2–7 µm and BEV entrapment efficiency was equal to 49%. Jiang et al. [22] developed innovative chitosan-PCL MPs loaded with BEV using the water-oil emulsion method. These MPs were studied in terms of cytotoxicity and injection feasibility and they could be considered as a potential carrier suitable for ocular delivery. Yandrapu et al. [23] prepared PLA nanoparticles into PLGA MPs loaded with BEV by using the supercritical infusion technique. The study reported a sustained release of the payload from the innovatively produced carrier. In addition, hydrogel particles have been proposed, such as alginate-based drug delivery systems [3] or alginate/chitosan polyelectrolyte complex [20]. Alginate is an interesting natural carrier because it can provide low interfacial tension with the surrounding biological environment and has a high capacity to absorb biological fluids; it is also characterized by a composition and mechanical properties that are similar to those of extracellular matrix. It has been reported that the encapsulation and release of monoclonal antibodies using an alginate hydrogel platform can significantly enhance their efficiency and targeting. However, the control of particle size distribution and hydrogel morphology is not simple.

From a bibliographic overview, it emerged that few studies reported a complete panel of physicochemical characterizations, including data of morphology, particle size distribution, polydispersity, and drug release kinetics, which are the most commonly considered basic parameters in the analysis of micro- and nanoparticulates. Moreover, there is a lack of well-consolidated in vitro toxicity studies with endothelial cells. Although these carriers have the potential to become new platforms for the therapy of vascular diseases, there is still a lack of knowledge regarding their applicability.

For this reason, the aim of this work was the production of BEV-loaded polymeric MPs, using PLGA, PCL, and PLA as carriers in order to compare the results obtained with these three different polymers in terms of particle size distribution, BEV encapsulation efficiency, and BEV release kinetics. In general, this work was aimed at defining a polymeric platform for the encapsulation of BEV to be useful in the treatment of angiogenesis-related diseases.

The water/oil/water (w/o/w) double emulsion technique was employed for the fabrication of PLGA, PCL, and PLA MPs that were characterized in terms of morphology, particle mean diameter, particle size distribution, entrapment efficiency, BEV release kinetic, granulometry at different storage temperatures (4, 25, and 37 °C), and in vitro validation with EA.hy926 human endothelial cells.

## 2. Materials and Methods

### 2.1. Chemicals

Poly(d,l-lactide-*co*-glycolide) (Resomer^®^ RG 505, Mw = 54,000–69,000 g/mol) 50:50 and poly(l-lactide) (Resomer^®^ L 207 S, Mw = 200,000–250,000 g/mol) were obtained from Evonik Industries (Essen, Germany), whereas poly(ε-caprolactone) (Mn = 80,000 g/mol), poly(vinyl alcohol) (Mw = 30,000–70,000 g/mol), and a solution of penicillin/streptomycin for cell culture were purchased from Sigma-Aldrich (Saint Louis, MO, USA). Ethyl acetate and chloroform, that were used as solvents to dissolve the polymers, Dulbecco’s modified Eagle medium (high glucose with L-glutamine and sodium pyruvate) (DMEM), fetal bovine serum (FBS), trypsin, and Dulbecco’s phosphate buffered saline (DPBS) were purchased from Carlo Erba (Milan, Italy). Bevacizumab (Avastin^®^, Genentech, Inc., South San Francisco, CA, USA) was kindly offered by the pharmacy of Ospedale Policlinico San Martino, Genoa, Italy. The QuantumMicroProtein bicinchoninic acid protein assay kit (Micro BCA) (Euroclone, Pero, Italy) was used in order to quantify BEV for the encapsulation efficiency and for the in vitro release studies. CellTiter 96^®^ AQ_ueous_ One Solution Cell Proliferation Assay (MTS) for cell viability studies was purchased from Promega (Madison, WI, USA).

### 2.2. Polymeric Microparticle Production

To produce PLGA-based MPs, the w/o/w technique was used. Briefly, 1.0 g of PLGA was dissolved in 19.0 g of ethyl acetate at room temperature (25 ± 2 °C) until its complete dissolution, then 800 μL of the aqueous inner phase was added. It consisted of a BEV solution (25 mg/mL) or DPBS for loaded or empty MPs, respectively. The first w/o solution was obtained by homogenization for 2 min (30 s on and 30 s off) using a Vibra-Cell^TM^ ultrasonic probe with 60% amplitude (Sonics & Materials, Inc., Newtown, CT, USA). After that, the first emulsion was added to 80 mL of previously prepared ethyl acetate saturated water. PVA was used as surfactant at 2% (*w*/*v*) in the aqueous solution [24]. The secondary emulsion was obtained using a rotor-stator emulsifier (Silverson L5T, Silverson Machines Ltd., Chesham, UK) at 7000 rpm for 6 min. Finally, the obtained emulsion remained under magnetic stirring at 300 rpm into a fume hood to obtain the complete evaporation of the solvent (about 3 h). Once produced, the particle suspension was washed with Milli-Q water three times by centrifugation at 12,984× *g* for 15 min at 4 °C (centrifuge from Alliance Bio Expertise MF-20R, Guipry, France) and the pellet was washed with deionized water to remove the excess of BEV.

**Figure 1:**
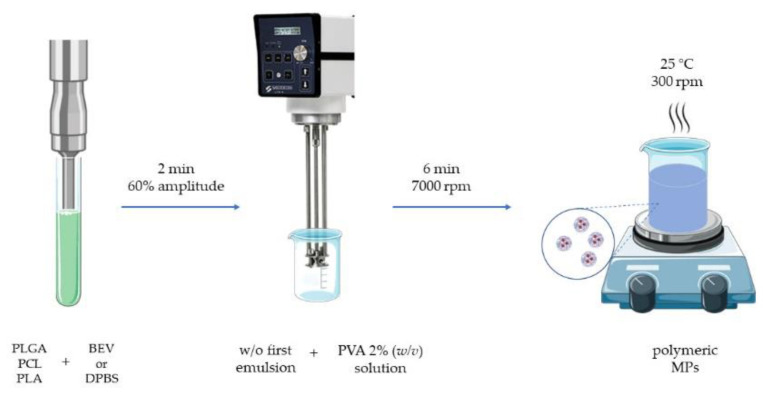
Schematic representation of MP production process. PLGA: poly(lactic-*co*-glycolic acid), PCL: poly(ε-caprolactone), PLA: poly(lactic acid), BEV: bevacizumab, DPBS: Dulbecco’s phosphate buffered saline, PVA: poly(vinyl alcohol), rpm: revolutions per minute, MPs: microparticles.

The PCL- and PLA-based MPs were produced using the same w/o/w technique but, in these two cases, chloroform was used as solvent. The same theoretical loading was fixed. The final w/o/w emulsion was prepared by adding the first w/o emulsion to 80 mL of the same PVA solution reported above in the dispersant phase. The emulsification step was performed using the rotor-stator emulsifier, as described before, at the same working conditions. The final emulsion remained under magnetic stirring at 300 rpm until the complete evaporation of the organic solvent was reached (Figure 1). The obtained particle suspensions were washed with Milli-Q water and centrifuged as described for the PLGA MPs. The obtained particles were stored at 4 °C until their use for the different experiments. Empty MPs with all the types of polymers were also produced and used as controls during the entire experimentation.

### 2.3. Microparticle Characterization

#### 2.3.1. Morphology Studies

The droplets formed in the emulsion were observed using an optical microscope (model BX50, Olympus, Tokyo, Japan), characterized by droplet size distributions, droplet mean diameter (DMD), polydispersity index (PDI), and span, and studied by laser diffraction method using a Mastersizer 3000 (Malvern Instruments Ltd., Malvern, UK). For all the samples, at least three replicates were analyzed. Additionally, particle suspensions were characterized in terms of particle size distribution (PSD), particle mean diameter (PMD), PDI, and span, using the same instrument. Suspensions were analyzed as obtained, without any dilutions.

The morphology of the produced MPs was observed using a high-resolution field emission scanning electron microscope (HR FE-SEM) (Carl Zeiss, Oberkochen, Germany). Samples were prepared for microscope observation by filtration on a 0.45 μm pore size filter paper (PRAT DUMAS, Couze-St-Front, France) and air-dried in order to recover dried MPs. The obtained powders were put on a stub and observed using gold metallization. HR FE-SEM parameters included an aperture size of 30 µm, an accelerating voltage equal to 7 kV, and a working distance of 7.7 mm. The electron microscope was mounted on a vacuum chamber equipped with a focused ion beam (FIB) (CrossBeam 1540 XB Carl Zeiss, Oberkochen, Germany), allowing for a more detailed investigation [25]. FIB is a column that generates and directs a stream of gallium ions focusing them onto the sample, both for the purpose of etching or milling the surface and as a method of imaging. Due to the ability of the FIB to mill material at micro- and nanoscale, the MPs were cross-sectioned in order to reveal the internal morphology. Using SEM coupled with FIB provides high spatial resolution with unique sample preparation in situ with no artifacts.

#### 2.3.2. Entrapment Efficiency

BEV entrapment efficiency (EE) was calculated by using an indirect method [26]. The quantity of free BEV was collected after centrifugation, quantified using Micro BCA, and the EE was determined using the following Equation (1):

(1) $$\mathrm{EE}\;(\%)=\frac{\mathrm{amount}\;\mathrm{of}\;\mathrm{initial}\;\mathrm{BEV}-\mathrm{amount}\;\mathrm{of}\;\mathrm{free}\;\mathrm{BEV}}{\mathrm{amount}\;\mathrm{of}\;\mathrm{initial}\;\mathrm{BEV}}\times 100$$

### 2.4. In Vitro Release Studies

The in vitro release tests were performed using all the polymer-based MPs. The formulations were suspended in 15 mL of DPBS (pH = 7.4) and stored at 37 ± 2 °C under constant stirring. At a pre-fixed time over a period of 7 weeks, samples were collected and replaced with the same volume of fresh DPBS. In order to calculate the BEV concentration, supernatants were analyzed by using Micro BCA. The obtained values were expressed as a percentage of released BEV over time and were determined as a cumulative release in terms of released mass of BEV at each time point. Release studies were performed in triplicate.

Kinetic models were employed to describe the drug release from the three polymeric formulations. Drug release from simple systems may be described by the well-known power law expression (Equation (2)) [27]:

(2) $$\frac{M_{t}}{M_{\inf}}{=\mathrm{Kt}}^{n}$$

where *M_t_* and *M_inf_* are the amounts of the drug released at time *t* and the overall amount released, respectively; *K* is a release constant; and *n* is a release exponent indicative of the release mechanism.

Classically, *n* = 0.5, 0.5 < *n* < 1, or *n* = 1, are indicative of Fickian release, anomalous transport, or case II transport kinetics, respectively. However, the *n* values may change with the matrix geometry.

The mathematical model proposed by Corrigan and Li [28], instead, is a more complex model that takes into account different possible release mechanisms, such as diffusion-controlled mass transfer and bulk degradation (Equation (3)):

(3) $$F_{\mathrm{tot}}{=F}_{D}{+F}_{\deg}$$

where *F_tot_* is the total fraction of the drug released at a given time, *F_D_* is the initial diffusion contribution of the drug that is accessible at the solid–dissolution medium interface (mass percentage) (Equation (4)), and *F_deg_* is the contribution of drug (mass percentage) entrapped in the polymer, whose release depends on the polymer bulk degradation.

(4) $$F_{D}{=F}_{\mathrm{Dinf}}\;(1-e^{K_{d}t})$$

The second component of Equation (3), *F_deg_*, is related to polymer bulk erosion and can be described by Equation (5):

(5) $$F_{\deg}{=(1-F}_{\mathrm{Dinf}})\left(\frac{e^{{\mathrm{Kt}-\mathrm{Kt}}_{\max}}}{{1+e}^{{\mathrm{Kt}-\mathrm{Kt}}_{\max}}}\right)$$

where *F_Dinf_* is the diffusion contribution at infinite time and *K_d_* is the first order constant associated with the diffusion-controlled release. *t_max_* and *K* are the time of the maximum drug release rate and the first order rate constant of drug release, respectively, mediated by polymer degradation.

### 2.5. Microparticle Biological Validation

EA.hy926 human endothelial cells (CRL-2922^™^, ATCC^®^, Manassas, VA, USA) were grown in DMEM supplemented with 10% (*v/v*) FBS and 1 % (*v*/*v*) penicillin/streptomycin and incubated at 37 °C and 5% CO_2_ until 70% confluency was reached. Then, cells were plated in 4 × 10^3^ per well. After 24 h, different concentrations (0.125, 0.250, 0.500, and 1.0 mg_BEV_/mL) of BEV-loaded PLGA, PCL, and PLA MPs were put in contact with the cells. An equivalent concentration of empty MPs for each type of polymer was used as control. In addition, even the same volume of the solvent (water) in which the MPs were suspended was used as reference for the experimentation (solvent). After 24, 48, and 72 h, MTS assay was assessed as reported by De Negri Atanasio et al. [29]. Briefly, 20 μL of reagent was added to each well and plates were incubated at 37 °C and 5% CO_2_. After 3 h, absorbance at 492 nm was read using a microplate reader (Tecan Spark^®^ 20M, Tecan, Männendorf, Switzerland). Control cells were considered to be 100% in respect to all the other samples. Experiments were done in triplicate.

### 2.6. Statistical Analysis

All the experiments were done in triplicate and the results are expressed as mean value ± standard deviation. Data were statistically analyzed by one-way analysis of variance (ANOVA), following Tukey’s HSD post hoc multiple comparison test by using Statistica v 8.0 software (StatSoft, Tulsa, OK, USA).

## 3. Results

### 3.1. Microparticle Production and Characterization

**Figure 2:**
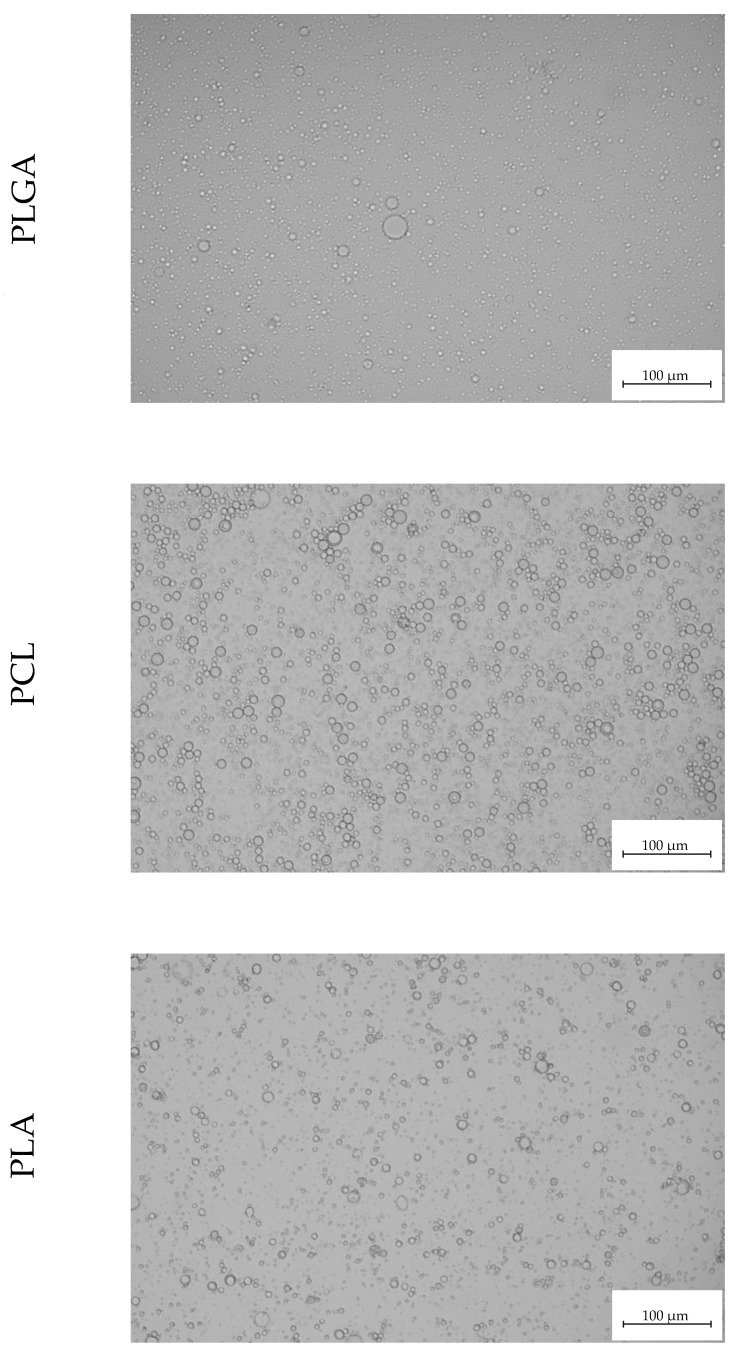
Representative optical microscope images of emulsions produced with the different polymers. PLGA: poly(lactic-*co*-glycolic acid), PCL: poly(ε-caprolactone), PLA: poly(lactic acid).

Empty and loaded MPs were produced by a double emulsion technique. Figure 2 shows optical microscope images of the emulsions obtained for the three types of the investigated polymers. Droplets were characterized by their dispersion and they resulted to be well-formed, not coalescent, and no instability phenomena occurred.

After solvent evaporation, the MPs were characterized in terms of DMD, EE, and PMD. Results are summarized in Table 1.

**Figure 3:**
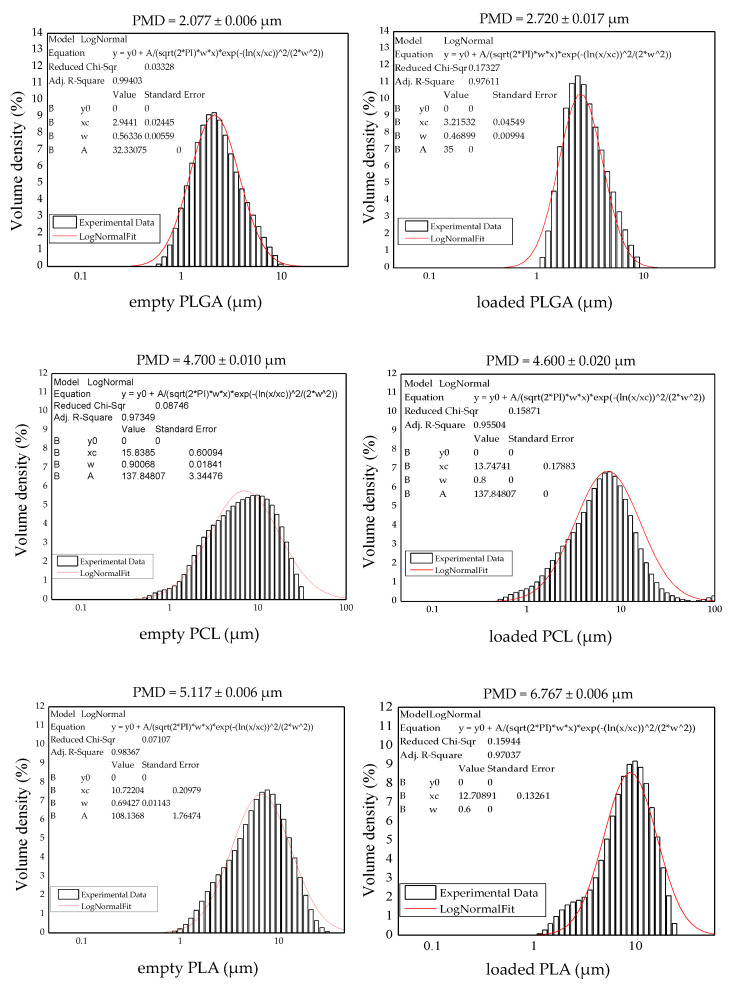
PMD and diameter distribution of MPs produced with the different polymers. PMD: particle mean diameter, PLGA: poly(lactic-*co*-glycolic acid), PCL: poly(ε-caprolactone), PLA: poly(lactic acid) both loaded and empty.

The DMD of the studied MPs significantly varied by using the three different polymers. In detail, it strongly depended on the molecular weight of the single polymer used for their production. It is well-known, in fact, that polymer molecular weight can affect oil phase viscosity and oil-water interfacial tension, resulting in larger droplets. The DMD was increased by using the three polymers in the same order of the molecular weight they possess, from PLGA, with the lowest, to PLA, with the highest. This trend was registered for both empty and loaded MPs (Figure 3). BEV encapsulation slightly affected the final DMD of loaded MPs. For both PLGA and PLA, an increase in the final PMD was a direct consequence of the antibody encapsulation (*p* = 0.0003). In contrast, in the case of PCL-based MPs, a decrease in PMD was observed. This difference caused by BEV encapsulation is probably due to the different chemical behavior of the two polymer classes used in this study. PLGA and PLA, in fact, are hydrophilic whereas PCL is hydrophobic.

BEV EE was high especially when using PLGA and PCL for which no differences were noticed. Considering the obtained results, it can be observed that larger particles entrapped less of the drug. This can be due to partial migration of the inner water phase towards the external water phase that was more pronounced for larger droplets that are also characterized by lower stability in emulsion.

**Figure 4:**
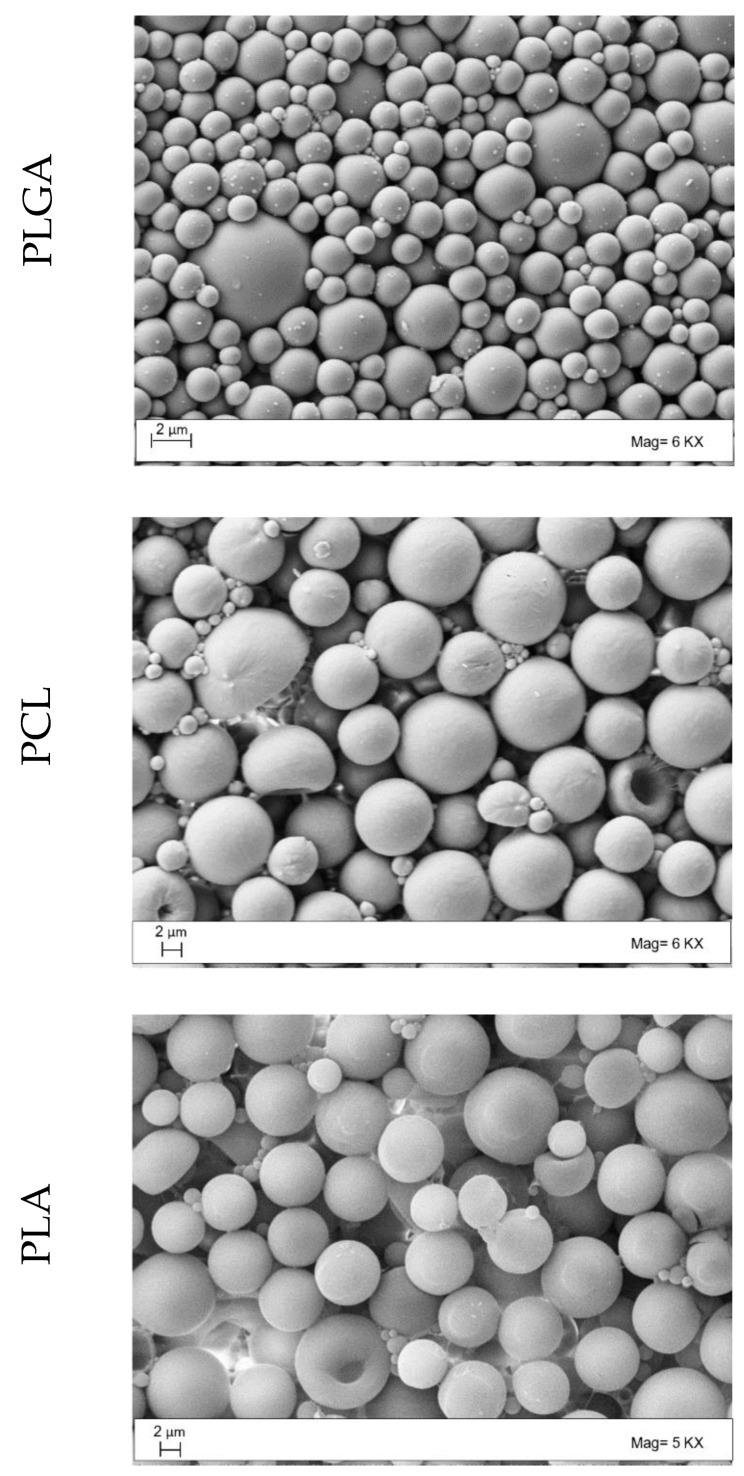
Representative HR FE-SEM images of the three different MPs. PLGA: poly(lactic-*co*-glycolic acid), PCL: poly(ε-caprolactone), PLA: poly(lactic acid).

Particle morphology was investigated using HR FE-SEM. Figure 4 reports HR FE-SEM representative images of the produced polymeric MPs. It can be observed that MPs were well-formed and spherical with a regular and smooth surface in all cases. Particle dimensions were in agreement with the results obtained with the PSD analyses.

**Figure 5:**
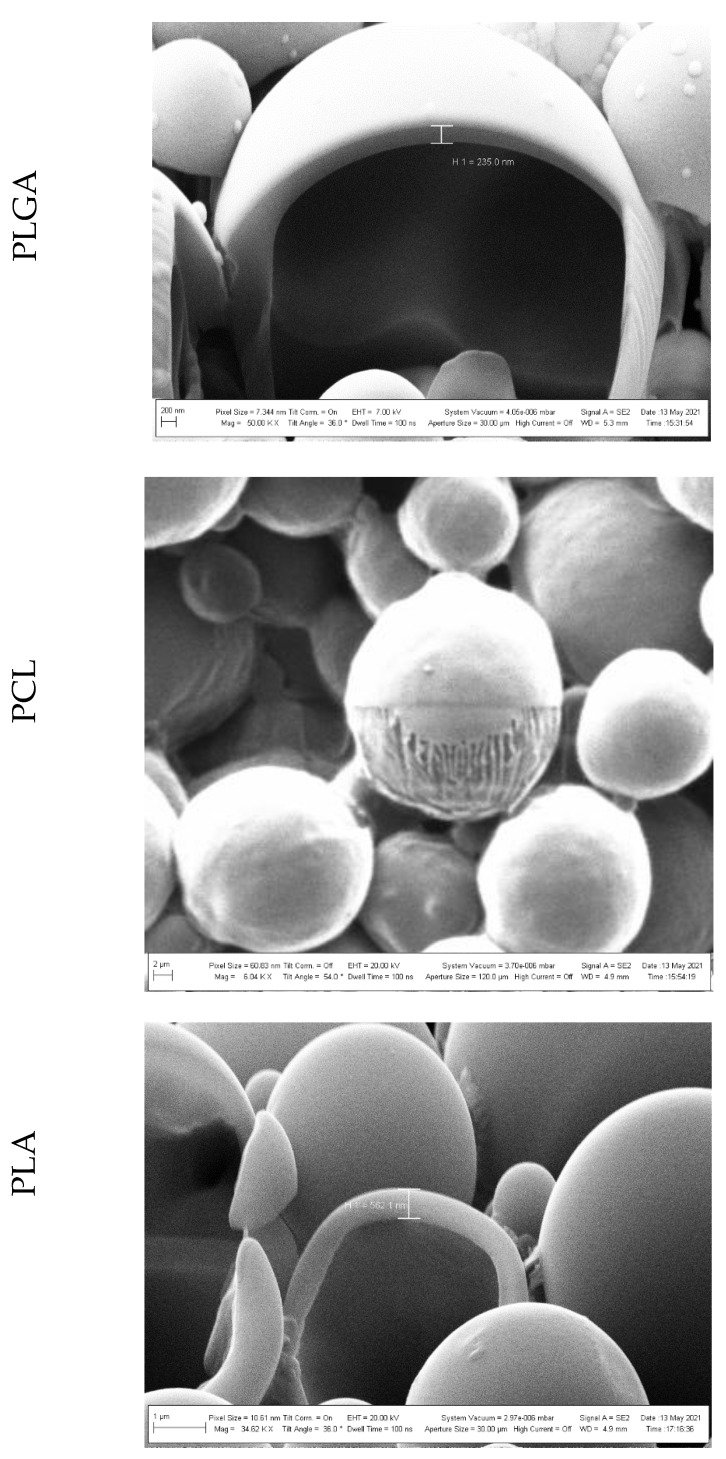
HR FE-SEM images of the cross-section of the three different MPs. PLGA: poly(lactic-*co*-glycolic acid), PCL: poly(ε-caprolactone), PLA: poly(lactic acid).

Particle internal morphology was also investigated using FIB. Figure 5 shows the cross-sections of the three samples obtained with a gallium ion energy of 30 kV and a ion current spanning from 10 pA to 50 pA, depending on the MP sizes.

From these images, it can be observed that PLGA and PLA MPs present a core-shell like structure, whereas for PLC MPs, a more compact structure filled with the polymer matrix was observed. No internal structure is visible in the FIB-sectioned MPs in the center of the HR FE-SEM image of PCL; the theatre curtain effect [30] on polymeric material that undergoes milling are due to a different sputtering yield at different incident angles. The measured shell thickness of the PLGA sample was around 250 nm, whereas for PLA it was almost double. It is clear from the images of PLGA and PLA that, in these cases, the cross-section did not introduce artifacts or modifications of the external structure.

### 3.2. Drug Release

**Figure 6:**
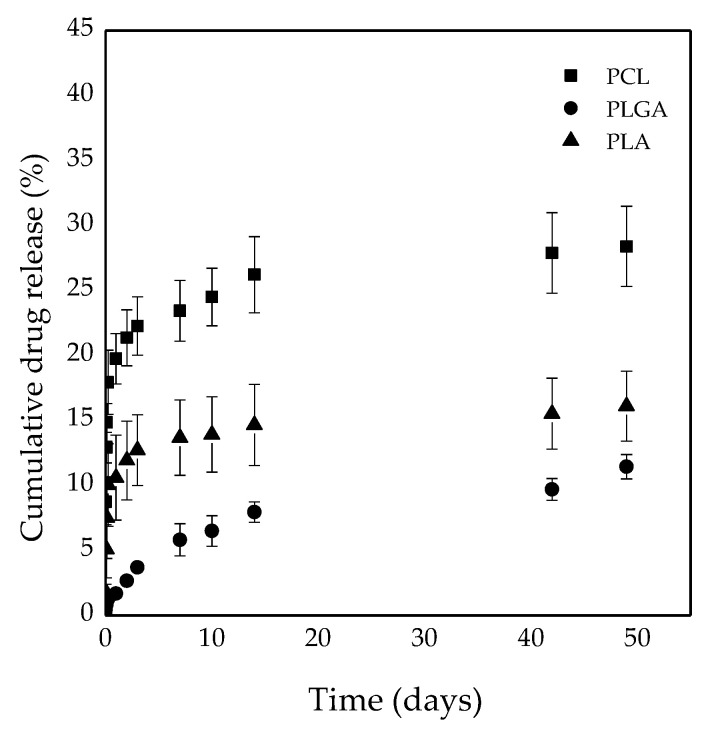
In vitro release profile for PLGA, PCL, and PLA MPs. PLGA: poly(lactic-*co*-glycolic acid), PCL: poly(ε-caprolactone), PLA: poly(lactic acid).

The release of BEV was studied for all the loaded polymeric MPs over a period of 50 days at 37 °C (Figure 6).

BEV was released in a sustained manner during the time of observation. In particular, each type of polymer affected the drug release rate. PLGA-based MPs showed the slowest release kinetic energy and no initial burst release was registered, indicating that almost all of the drug had been entrapped inside the particles without the presence of drug molecules on the polymer surface. BEV release from PLGA MPs was characterized by a constant release rate equal to 1.80 mg_BEV_/days. This behavior is typical of PLGA-based drug delivery systems, in which at first the drug is released by diffusion and then the erosion of the polymer accelerates the drug release. PCL MPs showed the fastest release in the first hours of observation, with an initial release of 20%. A sustained release was observed up to 50 days with an approximatively constant release rate of 14 mg_BEV_/days. PLA MPs showed a slower release, if compared to PCL MPs, but faster if compared to PLGA ones. Even working with these MPs, there was an initial release that corresponded to about 10% of the total cumulative released drug. The drug release rate amounted to 11 mg_BEV_/days.

At the end of the observation time, the cumulative release percentages were 11.92 ± 0.93, 28.38 ± 3.09, and 16.09 ± 2.68%for PLGA, PCL, and PLA MPs, respectively. The difference in BEV release can be attributed to the difference in morphological structure of the obtained MPs. Indeed, PLGA- and PLA-based MPs have the same behavior, characterized by limited initial burst release. For these two carriers, a core-shell structure was observed, in which all the protein was entrapped in the internal core and the release was observed after the diffusion of the protein through the polymer layer [31,32]. In the case of PCL, a compact dense structure was observed in which the protein is probably homogenously dispersed in the whole structure of the carrier, even at the surface [33]. For this reason, the protein present at the surface is rapidly released in the external medium by simple dissolution, inducing the first initial burst release, and then it is released by diffusion through the polymer matrix internal structure. Furthermore, the difference in release curves can be attributed by different crystallinities of the polymer used for particle preparation. Indeed, PCL is semi-crystalline, while PLGA and PLA are amorphous polymers [34,35]. Semi-crystalline polymers form porous structures, which release drugs more rapidly. The release from amorphous polymers is less rapid and more controlled by diffusion [36].

To better describe and understand the difference among the three MP systems, drug release data were described using two mathematical models, as reported in Section 2. In particular, one model takes into account the diffusion-controlled release (Equation (2)) and the other combines both the diffusion and the polymer degradation phenomena (Equation (3)).

**Figure 7:**
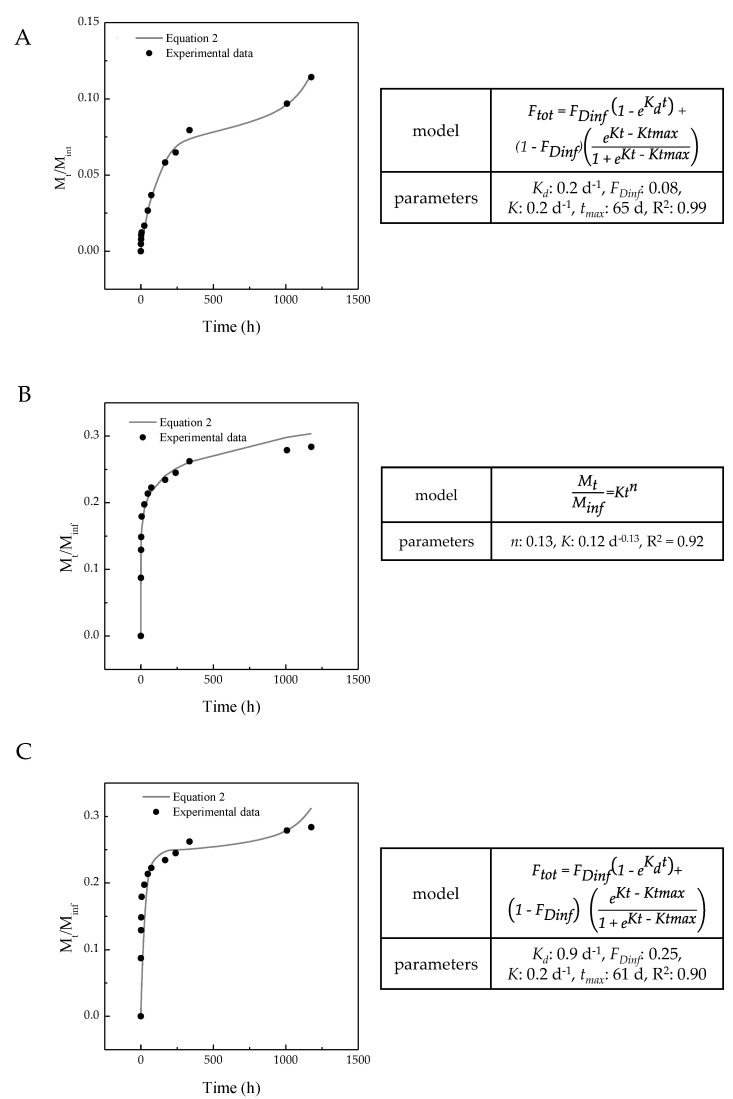
In vitro release profile fitting with the mathematical models from (**A**) PLGA, (**B**) PCL, and (**C**) PLA MPs. PLGA: poly(lactic-*co*-glycolic acid), PCL: poly(ε-caprolactone), PLA: poly(lactic acid).

A nonlinear least square fitting allows the estimation of the parameters *K* and *n* in Equation (2), and *K_d_*, *F_D_*, *K*, and *t_max_* in Equation (3). Drug release from PLGA and PLA MPs was better described by Equation (3), whereas drug release from PCL MPs followed Equation (2). In Figure 7, both the experimental points (dots) and model curve (line) are represented for the three drug release profiles that were studied. The values of the different parameters are reported in the same figure. The models give a fair representation of the drug release profiles, as can be seen from the continuous curves reported. Equation (2) provides an appropriate description of drug release from PCL MPs (R^2^ = 0.92); indeed, PCL belongs to the class of slowly degrading polymers, which takes more than 2 years to be completely degraded under physiological conditions. For this reason, drug release from PCL MPs was practically due only to burst and drug diffusion phenomena; indeed, after the initial burst and sufficient water penetration to the matrix, drug release proceeded to diffusion, which was the main mechanism that governed the release profile. Equation (3) was a better model for the description of drug release from PLGA and PLA (R^2^ = 0.99 and R^2^ = 0.90, respectively) MPs. In these cases, the drug release was governed both by diffusion and by polymer degradation.

### 3.3. Suspension Stability

The stability of PLGA, PCL, and PLA MPs was studied in terms of PMD and PDI at three different temperatures, 4, 25, and 37 °C, for four weeks. Stability data are reported in Table 2.

Considering the PLGA MPs, the diameter varied at lower temperatures, from 5.08 ± 2.43 μm at 4 °C to 2.72 ± 1.61 μm at 37 °C, whereas the PCL MPs did not present an important increase in terms of PMD. PLA MPs presented positive behavior with the PMD, which did not present any differences at all the tested temperatures.

### 3.4. Biological Validation

**Figure 8:**
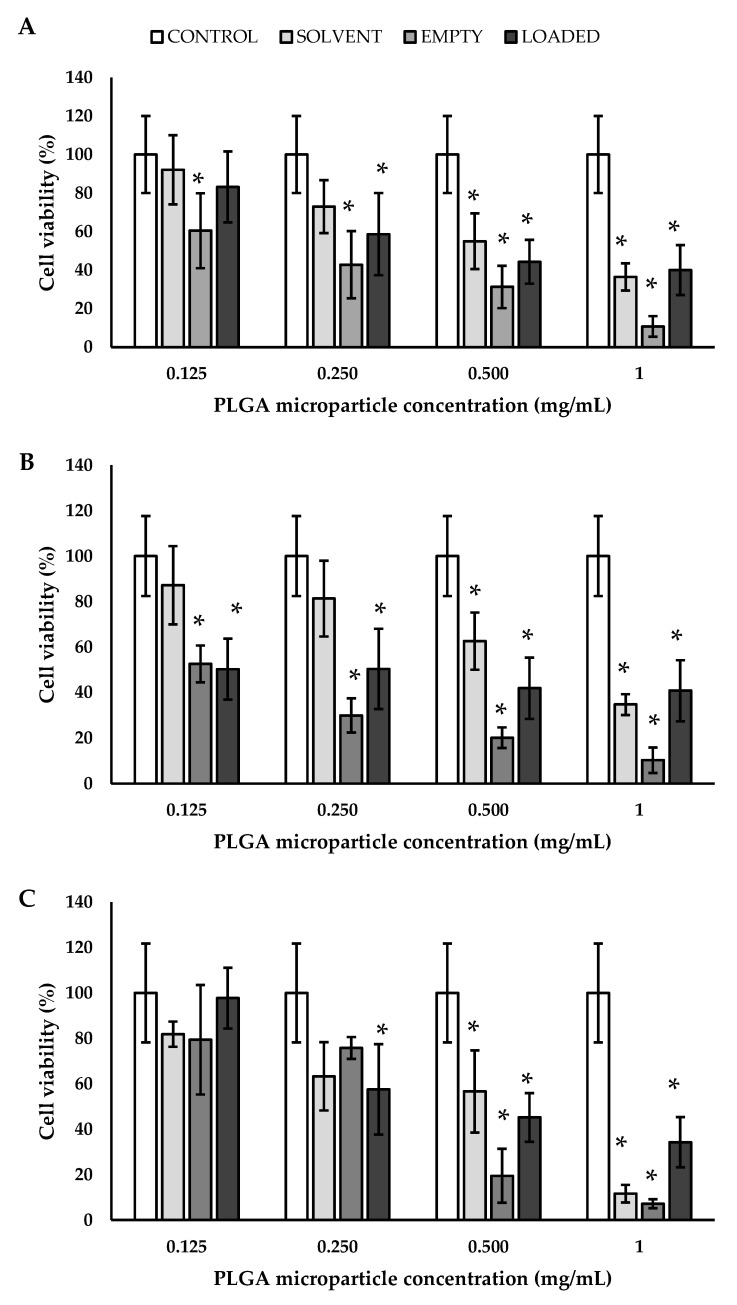
Cell viability of endothelial cells grown for (**A**) 24, (**B**) 48, and (**C**) 72 h in the presence of PLGA MPs, * *p* < 0.05 (with respect to the control using ANOVA with Tukey’s HSD post hoc multiple comparison test). PLGA: poly(lactic-*co*-glycolic acid).

**Figure 9:**
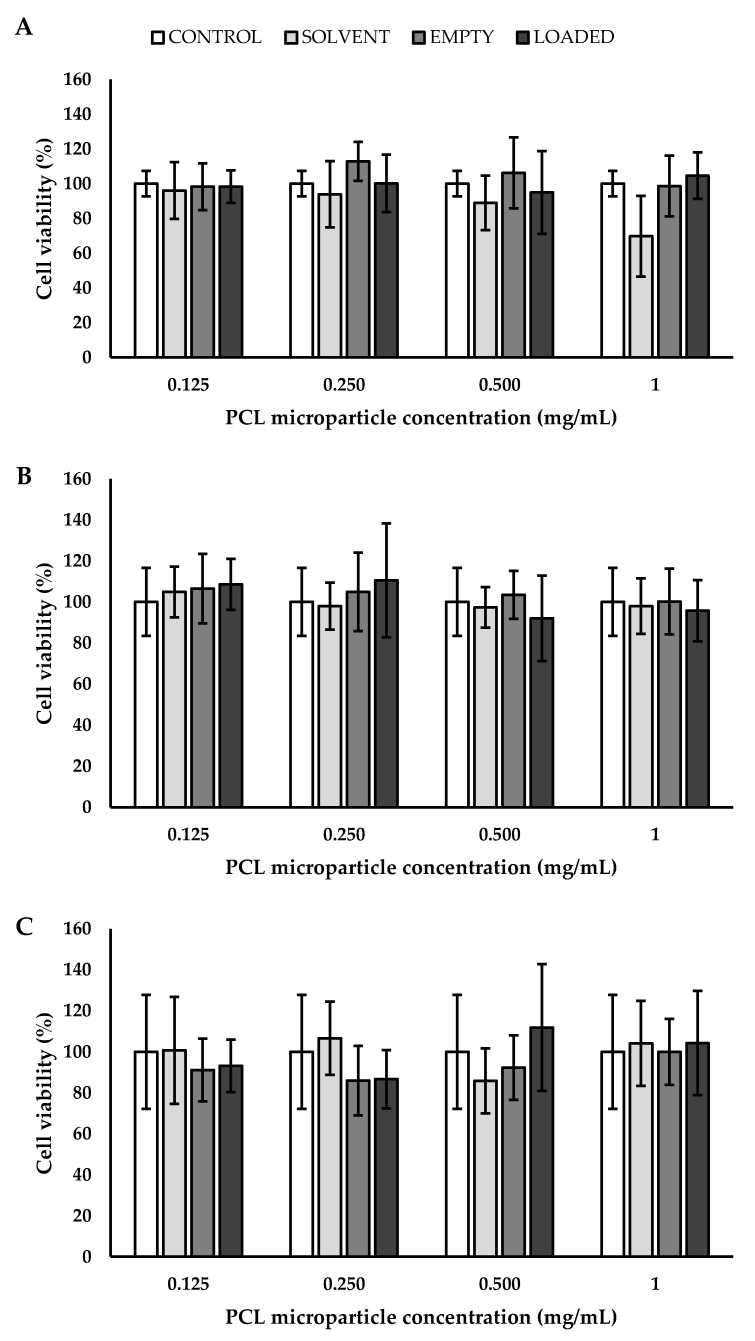
Cell viability of endothelial cells grown for (**A**) 24, (**B**) 48, and (**C**) 72 h in the presence of PCL MPs. PCL: poly(ε-caprolactone).

**Figure 10:**
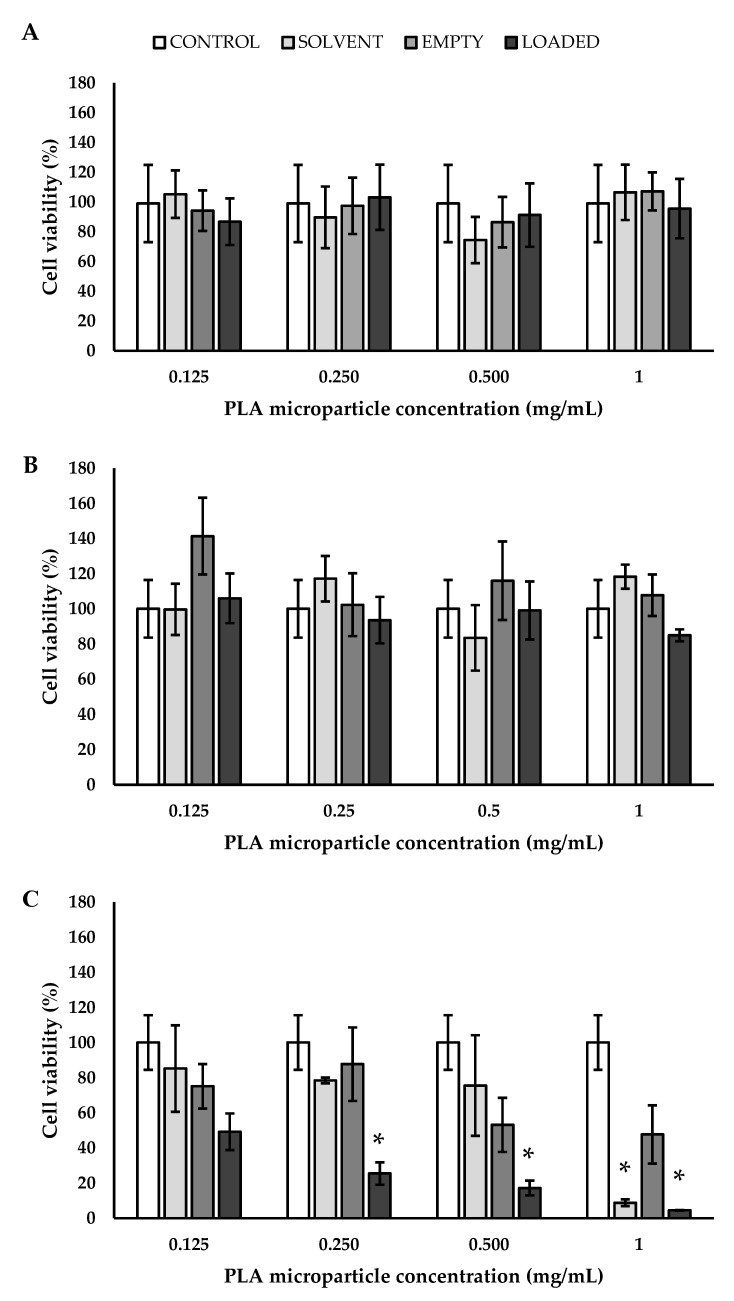
Cell viability of endothelial cells grown for (**A**) 24, (**B**) 48, and (**C**) 72 h in the presence of PLA MPs, * *p*< 0.05 (with respect to the control using ANOVA with Tukey’s HSD post hoc multiple comparison test). PLA: poly(lactic acid).

Before testing particle-dependent interactions with specific cell lineage, one important issue is always represented by understanding their compatibility with cells. Cytocompatibility is guaranteed by the employment of biocompatible polymers and by the use of non-toxic drugs, with a special attention paid to their concentration. In this work, PLGA, PCL, and PLA were used as polymers to encapsulate a monoclonal antibody, BEV. For this reason, these particles were tested on endothelial cells in order to biologically validate three platforms useful for all the applications where the blood stream is involved. Endothelial cells were chosen since they are intimately related to VEGF-A and, consequently, are a possible target of the encapsulated BEV. In detail, the metabolic activity of EA.hy926 was determined by measuring their mitochondrial functionality using MTS assay. Different concentrations of empty and loaded MPs were tested (0.125, 0.250, 0.500, and 1 mg_BEV_/mL) for 24, 48, and 72 h. As a control, solvent (water) was used since all the tested particles were produced by using it as a dispersant. Considering the cells grown in the presence of PLGA MPs (Figure 8), evidence of cytotoxicity was observed, even after 24 h. The reported cytotoxicity has to be ascribed partially to the low concentration of PLGA particles obtained in water solution and partially to the chemical nature of the employed polymer. In fact, even in the case of the control solvent, a partial cytotoxicity was put in evidence. A different trend was observed by working with PCL particles; taking into account the different days and the different concentrations, no statistical differences were noticed (Figure 9). It was observed that PLA MPs showed a partial cytotoxicity, especially in the presence of BEV, after 72 h of experimentation (Figure 10).

## 4. Conclusions

In this work, a comparative study among MPs made of three different polymers was completed PLGA, PCL, and PLA were used as polymers to encapsulate BEV in order to select the best tool for MP-based treatments intended for cardiovascular applications. In terms of size distribution, PLA particles showed a larger dimension while the use of PLGA guaranteed the production of particles of few microns in diameter and better control of PSD. PLA MPs showed the lowest EE value, being in any case acceptable for biomedical purposes. Different tests were performed in order to analyze the best polymer for BEV release. PLGA presented, compared to the other two polymers, higher EE and favorable stability at each studied temperature. Considering the release studies, PLGA MPs presented a controlled drug release for the designed time, which did not present an initial burst release.

The in vitro validation confirmed the biocompatibility of all the studied polymers, putting the attention on the maximum usable concentration for each one of them. PLGA-based MPs were characterized by partial cytotoxicity effects. Results obtained working with PCL-based MPs showed that, after 72 h, these MPs presented a good biocompatibility at all the studied concentrations. After 24 and 48 h, PLA-based MPs were biocompatible.

Considering the results obtained in the present work, it is possible to conclude that PLGA-based MPs offer the best properties desired for a drug delivery system, in terms of morphology, PMD, PDI, EE, and BEV release. Their cytocompatibility could be improved increasing the concentration of particles in the aqueous solution immediately after their production.

Future perspectives will regard the systematic study of the PLGA-BEV system in order to scale-down particle dimension to nanometric level, obtaining an injectable solution made of nanoparticles to be engineered in order to be able to target the atheroma.

## Figures and Tables

**Table 1:** DMD, EE of BEV-loaded MPs, and PMD of microcarriers prepared working with different polymers. Different letters (a, b, and c) reported in the DMD, EE, and PMD columns refer to statistically significant differences among the three different samples (empty or loaded) for each type of carrier (*p* < 0.05), using ANOVA with Tukey’s HSD post hoc multiple comparison test. Results are expressed as mean value ± standard deviation (*n* = 3). MPs: microparticles, DMD: droplet mean diameter, SD: standard deviation, EE: entrapment efficiency, PMD: particle mean diameter, E: empty, BEV: bevacizumab, PLGA: poly(lactic-*co*-glycolic acid), PCL: poly(ε-caprolactone), PLA: poly(lactic acid).

MPs	DMD ± SD (μm)	EE ± SD (%)	PMD ± SD (μm)
PLGA _E_	2.127 ± 0.006 ^a^	-	2.077 ± 0.006 ^a^
PCL _E_	4.090 ± 0.001 ^b^	-	4.700 ± 0.010 ^b^
PLA _E_	6.120 ± 0.066 ^c^	-	5.117 ± 0.006 ^c^
PLGA _BEV_	3.423 ± 0.025 ^a^	77.15 ± 0.36 ^a^	2.720 ± 0.017 ^a^
PCL _BEV_	4.437 ± 0.081 ^b^	76.54 ± 5.44 ^a^	4.600 ± 0.020 ^b^
PLA _BEV_	6.447 ± 0.064 ^c^	51.00 ± 0.28 ^b^	6.767 ± 0.006 ^c^

**Table 2:** PMD of PLGA, PCL, and PLA MPs stored at different temperatures. Different letters (a, b, and c) reported in the PMD column refer to statistically significant differences among the three different samples considered at the same temperature (*p* < 0.05), using ANOVA with Tukey’s HSD post hoc multiple comparison test. Results are expressed as mean value ± standard deviation (*n* = 3). MPs: microparticles, PMD: particle mean diameter, SD: standard deviation, PLGA: poly(lactic-*co*-glycolic acid), PCL: poly(ε-caprolactone), PLA: poly(lactic acid).

MPs	Temperature (°C)	PMD ± SD (μm)
PLGA	4	5.08 ± 2.43 ^a^
PCL		3.28 ± 2.23 ^b^
PLA		5.06 ± 1.20 ^a^
PLGA	25	4.32 ± 2.30 ^a^
PCL		3.69 ± 2.74 ^c^
PLA		5.08 ± 1.21 ^b^
PLGA	37	2.72 ± 1.61 ^a^
PCL		4.49 ± 3.29 ^b^
PLA		5.10 ± 1.19 ^c^

## Data Availability

Not applicable.
